# Ocular Manifestations of Alzheimer's and Other Neurodegenerative Diseases: The Prospect of the Eye as a Tool for the Early Diagnosis of Alzheimer's Disease

**DOI:** 10.1155/2018/8538573

**Published:** 2018-07-30

**Authors:** Pade Colligris, Maria Jesus Perez de Lara, Basilio Colligris, Jesus Pintor

**Affiliations:** ^1^Universidad Alfonso X, Madrid, Spain; ^2^Ocupharm Diagnostics SL, Madrid, Spain; ^3^Universidad Complutense de Madrid, Madrid, Spain

## Abstract

Dementia, including Alzheimer's disease (AD), is a major disorder, leading to several ocular manifestations amongst the elderly population. These visual disorders may be due to retinal nerve degenerative changes, including nerve fibre layer thinning, degeneration of retinal ganglion cells, and changes to vascular parameters. There is no cure for Alzheimer's, but medicines can slow down the development of many of the classic symptoms, such as loss of memory and communication skills, mood swings, and depression. The disease diagnosis is difficult, and it is only possible through PET scans of the brain, detecting evidence of the accumulation of amyloid and tau. PET is expensive and invasive, requiring the injection of radioactive tracers, which bind with these proteins and glow during scanning. Recently, scientists developed promising eye-scan techniques that may detect Alzheimer's disease at its earliest stage, before major symptoms appear, leading to improved management of the disease symptoms. In this review, we are discussing the visual abnormalities of Alzheimer's and other neurodegenerative diseases, focused on ocular functional-visual-structural biomarkers, retinal pathology, and potential novel diagnostic tools.

## 1. Introduction

AD is a degenerative disorder of the nervous system, eventually affecting over 10% of individuals aged 65 or over. It is the fifth-leading cause of death among people older than 65 years and a major cause of disability and poor health [1–4]. According to the World Alzheimer Report of 2016, it is estimated that 47 million people suffer from dementia worldwide, and this number could increase to more than 131 million by 2050, as populations age [5]. Life expectancy has increased dramatically over the past century across the globe and consequently has been detected an increase in the observed prevalence of chronic diseases among the elderly people.

While the optical examination sector appears extremely interesting for the development of a novel noninvasive diagnostic tool [6], genome-wide association studies have identified more than 20 AD risk genes [7–10], but the exact mechanism through which these genes are associated with AD remains unknown [11–13]. More promising are the studies on amyloid precursor protein (APP), presenilin 1 (PSEN1), and presenilin 2 (PSEN2) mutations, identifying them as important factors of autosomal-dominant early-onset AD and FAD [14].

As of 2018, there is no modifying therapy for AD [15], and only two families of medications are approved as palliatives for AD symptoms: the cholinesterase inhibitors and the N-methyl-D-aspartate receptor (NMDAR) antagonists, while various new drugs are under clinical trials evaluations, among them the promising new family of secretase inhibitors [16–18].

Even though many preclinical and clinical trials on AD drugs are underway, it is becoming obvious that the concept of the simplistic therapy of one compound against one target is failing, due to the complex pathophysiology of the disease, and future treatment will be based on drug combinations to intensify the results [19]. In any case, the onset of Alzheimer's disease cannot yet be stopped or reversed but might be manageable.

Diagnosis of the disease is difficult and frequently is subjective. There is no single diagnostic test and specialists are using various methods, including physical examination and brain scans, such as computed tomography (CT), magnetic resonance imaging (MRI), or positron emission tomography (PET), to rule out other probable causes for the symptoms [20]. For the moment, early diagnosis is based on the early recognition of the various signs and symptoms, as the most common sign of the disease is memory loss, especially forgetting recently learned information.

Generally, a timely and accurate diagnosis is considered to be important, as treatment of Alzheimer's and other dementia-causing diseases is typically most effective when started early in the disease process [21, 22]. Additionally, accurate diagnosis could transform the design and execution of clinical trials to test new AD treatments [23–27].

Current diagnosis methods mainly rely either on subjective tests or invasive methods with a limited accuracy. Definite AD diagnosis is possible only through postmortem observation of the aberrant accumulation of *β*-amyloid peptides (A*β*) extracellular aggregates, *β*-amyloid plaques, and protein tau intracellular twisted strands (neurofibrillary tangles, NFTs) [26–31]. The inaccuracy of the existing diagnostic methods, limits the general applicability for vast population screening and underlines the need for the identification of easily accessible tools for the identification of high-risk subjects. To better serve the population at risk of developing AD, new methods of definitive, noninvasive and lower cost diagnosis are needed.

AD could be categorized in familial Alzheimer's disease (FAD, also known as early-onset Alzheimer's disease) and sporadic type (also known as late onset Alzheimer's disease; LOAD) [32]. The eye offers a natural window to the brain as the retina, the light-sensitive layer lining the interior of the eye is considered part of the central nervous system (CNS) and the only optically accessible nervous tissue [33]. Neurodegenerative changes in the brain produced by AD are accompanied by structural and possible functional changes in the neuroretina and the ocular vasculature.

Numerous recent studies reported the presence of *β*-amyloid plaques in the retina of patients, opening the possibility of detecting AD though simple noninvasive eye scans [34–38]. In a parallel way, other studies revealed degenerative changes and retinal neuron damage in the retina/eye of AD animal models [39–43]. Consequently, various laboratories are trying to explore this approach, developing optical retinal imaging platforms capable to detect *β*-amyloid plaques in the retina of AD subjects [38, 44–48]. These novel imaging platforms are expected to early detect the presence of AD hallmarks and assist in monitoring the efficacy of probable future therapeutic agents which target relevant molecular pathways.

Nevertheless, Alzheimer's disease shares common features, mode-of-action, and targets with other neurodegenerative diseases such as, Parkinson's disease, Huntington's disease, amyotrophic lateral sclerosis, frontotemporal dementia, and even glaucoma and macular degeneration [49–52]. Therefore, in the following text, we will analyse the relation between AD and some of these neurodegenerative disorders, focusing on their ocular manifestations on the accessible retina, and the possibility of using these hallmark signs as structural-functional biomarkers for risk assessment-monitoring of disease progression, and evaluation for the drug therapeutic efficacy. In any case, the establishment of a link between neurodegenerative diseases and ocular manifestations is an interesting emerging field of study.

## 2. Detection and Pathophysiological Processes Involved in Alzheimer's Diseases and Other Neurodegenerative Diseases

Several studies have focused upon the relationship between neurodegenerative diseases and their possible ocular manifestation. There is compelling evidence to suggest that specific ocular biomarkers related to neurodegenerative disorders play a pivotal role in the development of retinal impairment or loss of visual function [53, 54].

The abnormal protein aggregation characterizes numerous neurodegenerative diseases, where insoluble proteins are accumulated in the brain such as AD, the Prion Encephalopathies, Parkinson´s disease, Huntington disease, and other polyglutamine disorders [55]. Protein misfolding diseases are not exclusive to the CNS, including disorders implicating amyloidogenic proteins like type 2 diabetes, inherited cataracts, vascular inflammation, atherosclerosis, and haemodialysis-related disorders, among many others [56]. Goldstein et al. reported the detection of nanoaggregates of *β*-amyloid in the human AD lens [57], a non-CNS structure considering another relevant ocular hallmark in this pathology. Deposits of *β*-amyloid are a distinct characteristic found in neurodegenerative diseases AD [58–60], Parkinson's disease [60–65], as well as being detected in age-related macular degeneration [66], and glaucoma [67–70].

In fact, there are numerous recent studies connecting glaucoma and AD, and some, bearing in mind the multiple common features to both diseases, including risk factors and pathophysiological mechanisms, are suggesting that Alzheimer's Disease and glaucoma should be considered age-related neurodegenerative diseases, coexisting in the elderly population [71–74]. Adding to the connection link theories between glaucoma and AD is the recent unifying hypothesis of the “glymphatic system.” This hypothesis is incorporating various aspects of the vascular, biomechanical, and biochemical theories, based on a few studies suggesting the existence of a paravascular transport system in the eye and optic nerve. However, for the moment, there are not sufficient available research data to totally support this idea [75–78].

On the other hand, recent studies are suggesting that AD and age-related macular degeneration (AMD) share common, pathological signalling defects and disease mechanisms at the molecular genetic level [79, 80]. There is growing evidence that *β*-amyloid, the main component of senile plaques, the hallmark of AD, is also a vital component of drusen, the hallmark of AMD [81–83]. While AMD is a retinal disease, AD is damaging the brain cells as well as the retina. These two age-related diseases are primarily affecting distinct parts of the central nervous system but are generally similar, concerning the abnormal extracellular deposits, metabolic and oxidative stress, neuroinflammation, and microvascular abnormalities [81, 84].

AD is characterized by visual disturbances at early stages which are correlated to neuronal damage in the inner retina, loss of RGCs, and optic nerve degeneration at postmortem histology, as suggested by several authors [37, 38, 85–91] and is also correlated to Parkinson's disease, as suggested by Bayer et al. [92]. Further studies reported that these inner retinal alterations were correlated with an abnormal pattern electroretinography (pERG) response [93]. From the earliest stages of the disease, amyloid plaque is deposited in retina tissue (including the RGC, retinal nerve fibre layer-RNFL, and inner plexiform layer) which can produce a fluorescence effect by using curcumin as a contrast [94].

An important characteristic of many systemic processes related to AD is the contribution of the energy metabolism, albeit the role of altered metabolic processes implicated in Alzheimer's disease pathogenesis is still unclear [95]. Recent studies discovered that in several CNS disorders, there is evidence of circulating metabolites signatures, suggesting that retinal oxygen metabolism in AD is slightly different from the normal, even though the results are not conclusive (ClinicalTrials.gov Identifier: NCT02253732; ClinicalTrials.gov Identifier: NCT00001972) [96, 97]. Following this discovery, the University of Iceland, Reykjavík, Iceland, is developing a biomarker for AD albeit further studies are necessary, using larger groups, to verify the results and further metabolomic studies and are imperative to demonstrate the correlation between systemic abnormalities and the brain impairment in AD [60].

The Spanish Hospital *Universitari Vall d'Hebron Research Institute* is investigating the association between type 2 diabetes (T2D) and Alzheimer's disease (AD) [98, 99] and in September 2014, started a clinical trial (ClinicalTrials.gov Identifier: NCT02360527) to explore whether diabetic patients are at risk of developing AD based on the assessment of retinal neurodegeneration and if these retina irregularities are related to the severity of AD. The results suggest that retinal sensitivity, assessed by microperimetry, is related to brain neurodegeneration and could be a useful biomarker for the detection of the disease [100].

In *the University Hospital of Bordeaux*, scientists are studying how the reduced thickness of RNFL relates to AD and other degenerative diseases and if RNFL thickness is associated with the development of cognitive impairment. They suggest that this parameter could be used as a biomarker for cerebral axonal degeneration, as RNFL deficits could be an early sign of AD, even before damages occur in the hippocampal region (ClinicalTrials.gov Identifier: NCT01621841- ClinicalTrials.gov Identifier: NCT01555827) [101–104]. The results confirm that the RNFL thickness is associated with changes in patients' memory and could be used as an indicator for the future development of AD [104].

In another instance, in the Department of Neurosurgery and Maxine Dunitz Neurosurgical Research Institute, Cedars-Sinai Medical Centre, Los Angeles, trials have begun to administrate intravenous therapy of curcumin, a fluorophore with high affinity to A*β*, to an AD-Tg mice (*n*=24) mouse model and performed fluorescent retinal examination in vivo [34]. After systemic administration of curcumin using this method, noninvasive optical imaging of retinal *β*-amyloid plaques in vivo with high resolution and specificity was allowed [105]. Curcumin is used as an early diagnostic probe due to its inherited fluorescence and high binding affinity to amyloid-*β*, but it could also be the basis of new drugs for the prevention and treatment of AD [106].

In 2011, Koronyo-Hamaoui and colleagues described the presence of *β*-amyloid in human retinal whole mount of AD. Posterior studies showed the predominant superior hemiretina damage involving mainly the inner retinal layers and histological analysis provided evidence of increased A*β*PP in AD retinas [5, 34, 49, 107]. In addition, specific retinal ganglion cells (RGC) and melanopsin retinal ganglion cells (mRGC) are affected by amyloid pathology in AD in and around cells [37].

Numerous studies of transgenic rodent models of AD have identified retinal A*β* depositions in the inner retina (inner plexiform layer, RGC layer, and nerve fibre layers) and the outer nuclear layer. These findings correlate with the burden of amyloid plaques in the brain [34, 38, 107–109]. Furthermore, the pathological mechanisms that involve *β*-amyloid in neuronal death are similar between RGC loss and neuronal degeneration in hippocampus in different models of AD [66, 85–90, 110]. RGC apoptosis was accompanied by and localized with A*β* deposits in retinas from rodent models, and signalling pathways are similar in retinal and hippocampal neurons death [107, 111]. Based on the observation on the retina of *β*-amyloid plaques, various companies and research centres started the development of specific biomarkers for the AD early diagnosis. In December 4, 2017 the Canadian company Optina Diagnostics Inc. started the ClinicalTrials.gov Identifier: NCT03420807 for the evaluation of a device called metabolic hyperspectral retinal camera (MHRC) for the detection of *β*-amyloid plaques. A hyperspectral mydriatic camera is used, producing multiple images of the retina, collecting and processing the light intensity for a large number (225) of contiguous spectral bands at high speeds allowing the noninvasive localization of structures and biomolecules in the retina evaluating their specific spectral signatures [112–114]. This method is based on the *β*-amyloid plaques hypothesis of AD pathophysiology, even though it generally remains unproven by clinical interventions [115].

Other studies support the hyperspectral imaging microscopy (HSI) method, to examine A*β* presence without the need for exogenous fluorophore. More and colleagues demonstrated the spectral signature of aggregated A*β* in AD mice model retinas and in vivo detection [116, 117].

While the optical examination sector appears extremely interesting for the development of a novel noninvasive diagnostic tool, for the moment, research has barely surpassed the animal model level, and there is limited proof yet that it is possible to detect *β*-amyloid plaques in the retina by visual means.

In addition to growing reports of retinal A*β* accumulation, other established hallmark of AD is pTau protein aggregates. Alterations in tau protein and gene expression were found in a triple transgenic mouse model revealing neuronal dysfunction that precedes cell death in the AD retina. In addition, these results were correlated to the presence of tau accumulation in the RGC soma and dendrites [38, 41, 118, 119]. Hyperphosphorylated tau was detected in postmortem human retinas and in AD animal models, which led us to the hypothesis that this could be observed at a presymptomatic stage of the disease as tau aggregation was not detected [118, 120, 121].

AD is a progressive brain disorder, with strong genetic determinants and aggregated protein, responsible for dementia. For many decades, the main environmental factors that influence the risk of AD development have been thoroughly studied. The primary risk factors involve hypertension, diabetes, hyperlipidaemia, metabolic syndrome, unhealthy diet, smoking, and physical inactivity among others [1, 122, 123]. It is considered that all these factors may contribute to the dilapidated physiopathological mechanisms in AD development.

Detection of specific biomarkers or advanced AD diagnosis approaches in the preclinical stage would be a potential breakthrough in the management of AD. Recent studies suggest that the retinal alterations occur early, thereby suggesting the necessity of earlier diagnosis and subsequent treatment of AD. Retinal imaging could, therefore, serve as an alternative approach to assessing brain degeneration in AD. Over the last 15 years, a noninvasive optical imaging technique of the retina and OHN has been used as a valuable tool to assess the optic nerve pathology in AD. In vivo detection of amyloid deposits in AD retinas were reported using OCT, and findings included perimacular and perivascular spots in the outer plexiform layer (OPL), ganglion cell layer (GCL), and the nerve fibre layer (NFL) thinning in AD [124–127].

In the Commonwealth Scientific and Industrial Research Organisation (CSIRO), Perth, WA, Australia, scientists began a study of AD patients, to discover whether the vascular analysis of retinal photographs could identify any retinal vascular parameters (RVPs) and when these retinal changes occur. The study demonstrated that retinal vascular biomarker is a potential method for the early detection of AD [128]. Likewise, researchers in Duke University of London developed a diagnostic method based on retinal imaging. In July 2017, a clinical trial with reference ClinicalTrials.gov Identifier: NCT03233646 was undertaken to evaluate a noninvasive OCTA-based retinal microvascular biomarker, as an effective screening tool in cognitive aging. The investigators hypothesize that microvascular network alterations in the retina mirror and possibly precede changes in the cerebral microcirculation seen in AD. Using advanced image analysis, the investigators aim to evaluate markers of reduced capillary blood flow and nonperfusion in the superficial and deep retinal vascular plexuses and choriocapillaris imaging using OCTA that would complement already established retinal-structural markers and increase their sensitivity and specificity in the early detection of AD. Various studies found that patients with AD have less density of retinal microvascular networks than the normal, considering retinal vessel reaction to flicker stimulation as a promising noninvasive, widely available, and easy-to-administer future biomarker for the diagnosis and monitoring of AD [129, 130]. In another study, the OCTA technique was tested on patients with AD, demonstrating its efficacy as a biomarker in the early diagnosis of the disease [131]. Additionally, the Medical University of Vienna is investigating whether more functional changes can be detected in the retina of AD patients, in conjunction with the already identified aberrations. For this purpose, in September 2016, they began a clinical trial (ClinicalTrials.gov Identifier: NCT02663531) evaluating the relationship between neural activity and blood flow, in induced flicker light hyperaemia in the retina, as a possible biomarker for AD. Furthermore, they assessed structural parameters such as retinal nerve fibre layer thickness and function parameters such as ocular blood flow and retinal oxygenation [132, 133]. According to the results, neuritic plaques were detected in representative cortical regions, suggesting OCTA as a useful tool for three-dimensional imaging of retinal A*β* accumulation [134, 135].

In general, advances in the image processing and software have improved the interpreting of anterior segment OCT images. In any case, the technique must advance towards the refinement of assessing vascular networks for the anterior segment and improvement of the histology detail, to become more accurate and to facilitate its use.

Studying the retinal visual changes of patients with neurodegeneration is an emerging field that requires more investigation. Intraocular and systemic therapeutic biomarkers/agents and visualizing techniques may be developed, serving the dual purposes of establishing novel pharmacological targets and new technological sources.

The mentioned areas of research have come a long way with many and important results, allowing us to have a better understanding of the disease [47]. More efforts are still needed, however, to fully understand the aetiology of the disease, to establish reliable individual predictive models, and place us closer to the development of a definitive diagnostic method and a possible personalized treatment.

## 3. Ophthalmological and Ocular Clinical Symptoms

The ocular alterations in AD involve different structures in the anterior and posterior pole (Table 1). In the anterior pole, tear fluid is considered to be a potential biomarker source. Kallo et al. discovered 100% increase of flow rate and 100% increase in protein levels in AD patients, with respect to the age-matched controls. The combination of four tear proteins lipocalin-1, dermicidin, lysozyme C and lactritin, had 81% sensitivity and 77% specificity for AD [136]. Furthermore, abnormal tear functions and reduced corneal sensibility were described in previous studies of neurodegenerative diseases [137]. In 2017, the French company Amoneta Diagnostics SAS started a clinical trial (ClinicalTrials.gov Identifier: NCT03030586) to validate the performance of a noninvasive peripheral blood diagnostic kit for AD, collecting samples from peripheral body fluids, tears being among them.

Pupillary responses are associated with brain impairment in different studies where light reflex amplitude is reduced in AD [177]. Granholm et al. suggest that task-evoked pupillary responses may be a psychophysiological biomarker of early risk for AD [158]. The Massachusetts General Hospital started a clinical trial (ClinicalTrials.gov Identifier: NCT03330353) in 2017 to investigate rod, cone, and melanopsin-driven pupillary light response in individuals, as a possible AD and other neurodegenerative diseases diagnostic method. Furthermore, the Centre Hospitalier Universitaire de Besancon France recently began a clinical trial (ClinicalTrials.gov Identifier: NCT01434940) to validate eye movement recording as an early differential diagnostic tool for AD. Tracking eye movements is becoming an interesting diagnostic technique and could be beneficial in recognizing individuals who will progress to AD, but for the moment, there remain many critical points which require additional investigation.

As previously commented, *β*-amyloid was detected in the human lens of AD patients, and in a Framingham Offspring Eye Study cohort, a correlation between cortical cataract and AD degeneration was observed [124, 156, 178, 179]. These findings establish the *β*-amyloid protein as a key pathogenic biomarker between lens and AD. In 2017, the American company Cognoptix, Inc. started a clinical trial to detect *β*-amyloid in the lens of the eye in patients with mild cognitive impairment (MCI), and mild AD after the instillation of Aftobetin Hydrochloride. Aftobetin, also known as ANCA11 and NCE-11, is an amyloid-binding compound applied topically in the form of an ophthalmic ointment and may be useful as an aid in the diagnosis of AD (ClinicalTrials.gov Identifier: NCT02928211).

Finally, in another ocular fluid, the aqueous humour; higher levels of proteins involved in AD pathology such as *β*-amyloid peptides; and a series of chemokines, including eotaxin, eotaxin 3, and interleukin [IL]-8 (160) were detected in cataract surgery patients. Additionally, Inoue et al. suggest that open-angle glaucoma patients presented elevated levels of various biomarkers of AD in the aqueous humour, including apolipoprotein (Apo) AI, ApoCIII, ApoE, transthyretin (TTR), complement factor H, complement C3, and *α*2-macroglobulin (*α*2M) [162].

In the posterior pole, retinal thickness is correlated to cortical atrophy and choroid and NFL is thinning in AD patients [85, 93, 144, 145, 153, 155, 160]. Several reports in AD patients, showed RGC loss [85, 86, 143]. Additionally, studies in patients and on AD animal models displayed A*β* and tau proteins accumulation in inner retinal layers [138, 143, 180]. Furthermore, Rusu et al. detected APP metabolism impairment in transgenic drosophila [140] and Paris et al. detected a reduction of angiogenesis processes in transgenic mice [151]. In addition, studies on patients and animal models reveal that activated microglia and proinflammatory molecules are contributing to the development of the pathology, and they are degenerating neurons, decreasing retinal activity [111, 148, 149, 181].

All these morphological and/or biochemical changes are leading to decreased visual acuity, deficits in visual perception, anomalies of colour vision, visual cognition impairment, and visuospatial disorientation. In summary, all these findings are creating the certainty that visual abnormalities of the visual axis, from RGC to visual cortex, are common ocular characteristic features of AD [164–166, 168].

## 4. Conclusions

AD diagnosis is based on the interpretation of the signs and symptoms of the disease in cerebrospinal fluid (CSF) tests and neuroimaging of the brain. Recent major advances in brain imaging are providing opportunities for a more accurate diagnosis, but unfortunately, this technique has limited availability, and its costs are high, making population-wide screening challenging. Consequently, there is a global need for novel early diagnostic methods, more specific, more economic, and with the added ability to distinguish AD among the diverse types of neurodegenerative disorders, bearing in mind that AD diagnosis must identify the disease before the cognitive deficits have reached the threshold of dementia.

The eye shares many neural and vascular similarities with the brain, directing many researchers towards the discovery and development of novel ocular biomarkers, which could have a profound influence on early diagnosis and the discovery of new treatments for the disease.

Numerous recent studies reported the presence of *β*-amyloid plaques, the hallmark of the disease, in the retina of AD mice models and humans, opening the possibility of detecting AD through a simple noninvasive eye scan. Various laboratories are exploring this approach with the development of optical retinal imaging platforms to detect *β*-amyloid plaques in the retina of AD subjects. These novel imaging platforms are expected to help aid the early detection of AD and assist in monitoring the efficacy of probable future therapeutic agents which target relevant molecular pathways.

Another imminent change concerning the diagnosis of the neurodegenerative diseases through the eye would be the greater role of the primary eye care practitioners in a sector that previously had little implication.

While a considerable number of clinical trials are currently underway to evaluate novel diagnostic methods, through the eye, and the results are promising, for the moment, the biomarkers for the diagnosis of Alzheimer's disease (AD) are not yet validated for use in clinical settings, and their widespread implementation remains a challenge. Among the reasons for this, we could underline the lack of experience of the researchers to interpret the huge data obtained from these eye images, ethnic variation causing further complications, the overlap with other diseases, and the lack of specificity of these biomarkers. In general, there is a need for a greater number of validation studies involving wider cohorts of various ethnicities and origins. Another question arising in the use of eye biomarkers is that perhaps they are too late as by the time *β*-amiloide plaques are discovered in the retina, the disease has already progressed.

Finally, the cascade of events resulting in retinal cell death in ocular neurodegenerative pathologies is leading scientists to a new common descriptive term of diseases of the retina. Even though the aetiology of AMD, glaucoma, and AD is still uncertain, studying the common features between them may provide further knowledge about their pathogenesis and could lead to accelerated development of therapies for both diseases. Additionally, studying AD-related retinal degeneration is a promising way forward in the investigation of AD pathologies and therapies, which could eventually benefit the brain and cellular mechanisms in other retinal degenerations.

## Figures and Tables

**Table 1:** Visual system pathological and functional alterations in Alzheimer's disease.

Ocular structures and clinical facts	Pathological changes
Retina	Deposition of proteins tau, A*β*, and pTAu [35, 38, 39, 118]; accumulation of phosphorylated tau protein [138, 139]
	Impairment of amyloid *β* precursor protein (APP) metabolism [140, 141, 142]
	Reduction in retinal ganglion cells (RGCs) [85–87,143]
	Reduction of retinal thickness [85, 93, 144]
	Reduction to the retinal nerve fibre layer (RNFL) thickness [89, 145]
	Retrograde degeneration from loss of cortical neurons [146, 147]
	Inflammation [148, 149, 150]

Retinal and choroidal vasculature	Retinal and choroidal vascular *β*-amyloid deposits in transgenic mouse model of AD lead to retinal degeneration [107]
	Reduction of vascularization [151]

Retinal vascular blood flow	Blood flow abnormalities may cause neurodegeneration [152]

Optic nerve	Axonal degeneration in the axonal segments [85, 140]
	Loss of optic nerve thickness [153, 154]
	Papillary paleness due to axonal loss and perfusion alterations [155]

Lens	Correlation between AD and supranuclear cataract [156, 157]
	Presence of abnormal protein deposits [124, 157]

Tears	Changes in the chemical barrier composition of tears [136]
Cornea	Reduced corneal sensitivity [137]

Pupil	Pupillary responses possible biomarker [158, 159]
Choroid	Attenuation of choroidal thickness [160, 161]

Aqueous humour	Detection of Alzheimer connected, *β*-amyloid peptides, and chemokines in the aqueous humour [162, 163]

Visual field	Dysfunction in different tasks of basic vision and visual cognition [164]
Visual acuity	Decreased visual acuity [165]

Sensory perception	Clinically important symptoms of visuospatial disorientation [166, 167]
Visual processing	Deficits of visual motion perception [168]

Contrast sensitivity	Contrast sensitivity disturbances and motion perception [169, 170]
Colour vision	Incomplete achromatopsia [171]

Stereopsis	Reduced stereoscopic depth perception [172, 173]

Circadian rhythm	Alterations in the circadian rhythm and *β*-amyloid deposits inside and surrounding degenerating mRGCs [174–176]
